# Large Scale Brain Functional Networks Support Sentence Comprehension: Evidence from Both Explicit and Implicit Language Tasks

**DOI:** 10.1371/journal.pone.0080214

**Published:** 2013-11-11

**Authors:** Zude Zhu, Yuanyuan Fan, Gangyi Feng, Ruiwang Huang, Suiping Wang

**Affiliations:** Center for Studies of Psychological Application and School of Psychology, South China Normal University, Guangzhou, China; University of Pennsylvania, United States of America

## Abstract

Previous studies have indicated that sentences are comprehended via widespread brain regions in the fronto-temporo-parietal network in explicit language tasks (e.g., semantic congruency judgment tasks), and through restricted temporal or frontal regions in implicit language tasks (e.g., font size judgment tasks). This discrepancy has raised questions regarding a common network for sentence comprehension that acts regardless of task effect and whether different tasks modulate network properties. To this end, we constructed brain functional networks based on 27 subjects’ fMRI data that was collected while performing explicit and implicit language tasks. We found that network properties and network hubs corresponding to the implicit language task were similar to those associated with the explicit language task. We also found common hubs in occipital, temporal and frontal regions in both tasks. Compared with the implicit language task, the explicit language task resulted in greater global efficiency and increased integrated betweenness centrality of the left inferior frontal gyrus, which is a key region related to sentence comprehension. These results suggest that brain functional networks support both explicit and implicit sentence comprehension; in addition, these two types of language tasks may modulate the properties of brain functional networks.

## Introduction

Sentence comprehension is the key step moving from word recognition to higher level language abilities. Sentence comprehension constructs coherent utterances from very basic semantic blocks such as word and phrase information [1], [2]. When subjects are asked to explicitly comprehend a sentence, such as when making a semantic congruency judgment, widespread functionally separate brain regions [3], [4] are integrated into a functional network that supports sentence comprehension. These regions include the left middle temporal gyrus (MTG), which is responsible for representation retrieval [5]; the left anterior temporal lobe [6]–[8] and the left angular gyrus [9], which are responsible for syntax and semantic interactions; the left posterior superior temporal gyrus (STG) [10], [11] and left inferior frontal gyrus (IFG) [12]–[16], which are responsible for sentence-level integration; and the cingulate cortex [17], [18], which is responsible for semantic control processing. Functional connectivity analyses have revealed that the inter-regional couplings between the frontal and temporal regions, as well as between the frontal and parietal regions listed above, are recruited in explicit sentence comprehension [19]–[21].

In contrast to widespread brain activation in explicit language tasks, neuroimaging studies using implicit language tasks have found brain activation in fewer regions of the frontal and temporal cortices. For example, in a study of sentence comprehension, Crinion et al. [6] found that temporal regions and a small portion of the left inferior frontal gyrus was active during an implicit language task, while Scott et al. [22] found that only the left superior temporal sulcus was recruited. Similarly, we found that sentence comprehension in an implicit language task elicited activation only in the left inferior frontal gyrus when increasing the difficulty of sentence comprehension [15].

These discrepancies may result from differences between various language tasks. Previous studies of sentence comprehension found that explicit language tasks may involve task-specific strategy effects [17], [23], [24]. For example, when subjects are asked to make congruency judgments regarding semantically congruent and incongruent sentences, they may need to monitor for semantic incongruence; however, this may not be necessary for sentence comprehension. This type of task may also involve attentional control [25], which is supported by activity observed in the frontoparietal attention network [26]. Thus, brain activation patterns in explicit language tasks may be confounded by effects specified by the task itself. In our recent studies [12], [27], a font size judgment task was used as the implicit semantic task for comparison with an explicit language task. Given that both the explicit and implicit language tasks engage sentence comprehension, a conjunction analysis of the two tasks would effectively reduce brain activations associated with task-specific strategies; in addition, this would provide a description of the neural basis of semantic activation. Indeed, we found restricted activation in the inferior frontal gyrus (IFG) using a conjunction analysis [12].

Considering that the functionally segregated regions were integrated into a functional network to support sentence comprehension, we believe that the differences in activation observed between explicit and implicit language tasks raise at least two questions. One question is whether strategy effects can confound brain activation patterns or if similar large brain networks will be active in implicit language tasks as in explicit language tasks. The other question is whether explicit and implicit language tasks can modulate the properties of brain functional networks. By using an implicit language task, Spitsyna et al. [28] found processing streams including the left STS, temporal pole, fusiform gyrus (FFG), temporo-occipital junction as well as the parietal cortices in semantic processing. However, as within-subject task comparisons are lacking, little is known regarding whether the brain networks recruited in explicit and implicit language tasks are the same, or how language tasks modulate the functional networks.

In this study, we used a graph theoretical approach which has been widely used for characterizing brain network architectures [29], [30] to compare the topological properties of the brain functional networks involved in explicit and implicit language tasks [12]. In the explicit task, subjects were asked to make a semantic congruency judgment on sentences. In the implicit language task, subjects were asked to make font size judgments during which their attention was not necessarily orientated to semantic processing. We assumed that the brain functional networks would fit small-worldness criteria regardless of task-specific stimuli. We also expected that the shared active regions in sentence comprehension during explicit and implicit language tasks would be identified as hubs of the functional networks, and the high local and global efficiency of the network can help us to infer the functional integration and segregation, which are two major organizational principles of the human brain [31]. The explicit language task required elaborated semantic analysis, which included different sub-processes, such as semantic retrieval and semantic integration. As these processes involved widespread regions of the entire brain, the explicit language task may thus emphasize global information transfer and alter the properties of the key regions in sentence comprehension, such as the left inferior frontal gyrus (IFG).

## Methods

### Subjects

We recruited twenty-seven healthy native Chinese speakers for the present study. All participants were right-handed with normal or corrected-to-normal vision. None had a current or past history of any neurological disorder or brain injury. To obtain acceptable data, five participants were removed because the top part of the cortex was not imaged in the MRI scan, and another was removed due to head motion artifacts larger than 2 mm. Thus, twenty-one subjects were retained (12 F/9 M, aged 19–28 years, mean ± *SD*  =  22±2.2 years) for further data analysis. Written informed consent was obtained from each subject and this study was approved by the Ethics Committee of Shenzhen People's Hospital, Shenzhen, China.

### Stimuli

To manipulate the difficulty of sentence comprehension, we constructed three versions of sentences: high cloze (HC) sentences, low cloze (LC) sentences, and violation sentences (SV). For the HC sentences, we first constructed a base set of 216 sentences with high constraint contexts. Constraint refers to the probability that the reader will expect a particular word in a given context. As illustrated in Table 1, each of the HC sentences was then modified to produce a LC sentence by replacing the selected noun with an unexpected noun, which was semantically congruent with the context of the sentence. A SV sentence was constructed by replacing the critical word of a HC sentence with a word that was incongruent with the context of the sentence. We thus constructed three versions of each sentence that differed from each other by only one critical word. Each sentence contained 11 Chinese meaning blocks/words. In half of these sentences, the critical word occurred in the 6^th^ position. In the other half of these sentences, the critical word occurred in the 7^th^ position. The critical words were matched by frequency (mean and standard deviation, HC: 23.7±47.1; LC: 23.6±58.1; SV: 21.1±50.4; *F* < 1) [32] and visual complexity (mean stroke number and standard deviation, HC: 8.1±2.3; LC: 7.9±2.3; SV: 8.0±2.1; *F* < 1) across the three versions of each sentence.

**Table 1:** Example stimulus materials used in the explicit and implicit language tasks in the present study.

Condition	Sentence
Example 1	
HC	医生成功地为病人做手术使病人及时得到治疗。
	The patient got treatment in time after the doctor successfully finished the **surgery**.
LC	医生成功地为病人做诊断使病人及时得到治疗。
	The patient got treatment in time after the doctor successfully finished the **diagnoses**.
SV	医生成功地为病人做轮胎使病人及时得到治疗。
	The patient got treatment in time after the doctor successfully finished the **tires**.
Example 2	
HC	由于伤病刘翔错过了机会让人们都十分惋惜。
	The people felt sorry for Liu Xiang as he missed the **game** due to disease.
LC	由于伤病刘翔错过了机会让人们都十分惋惜。
	The people felt sorry for Liu Xiang as he missed the **chance** due to disease.
SV	由于伤病刘翔错过了判断让人们都十分惋惜。
	The people felt sorry for Liu Xiang as he missed the **judgment** due to disease.

Note: Three types of sentences, high cloze (HC) sentences, low cloze (LC) sentences, and violation sentences (SV), were adopted to manipulate the difficulty levels of the sentence-level semantic unification in both the implicit and explicit language tasks.

The contextual constraint of each sentence was rated by thirty-three subjects who did not participate in the cloze test but were selected from the same subject pool used to select those who participated in the cloze test [33]. During each rating, the sentence stems up until the critical word was presented, and the subjects were asked to continue the sentence with the first noun that came to mind to make the sentence meaningful. The rating results were as follows: the mean cloze probability was 63.5% (range  =  30 – 100%, SD  =  19.3%) for the HC sentences, while the values of cloze probability were around zero for both the LC and SV sentences.

The semantic acceptability of each sentence was rated by another twenty new subjects from the same subject pool as used in the final experiment. A 5-point scale in which 1 stood for extremely unacceptable and 5 stood for completely acceptable was used for this purpose. The average semantic acceptability ratings were 4.3±0.3, 4.2±0.3, and 1.6±0.4 for the HC, LC, and SV sentences, respectively. Statistical analyses revealed a significant main effect of semantic acceptability in the three versions of sentences (*F* (2,430)  =  5028, *p* < 0.001), and all of the following pair-wise comparisons differed significantly from each other (HC > LC > SV, *p*-values < 0.001).

Sentence presentation was counterbalanced across tasks and participants in two steps. First, the 216 sets of sentences were divided into three groups, each containing 72 sets of sentences. One group was used for a semantic congruency judgment (SEM) task, another group was used for a font size judgment (FONT) task, and the third group was used for a silent reading (READ) task. MRI scans between these groups and tasks was counterbalanced between participants as well. The second control step involved counterbalancing the three sentence conditions in each task between participants. Therefore, each sentence frame would be presented to each participant only once without any repetitions. For each task, each subject was presented 24 HC sentences, 24 LC sentences, and 24 SV sentences. Within each task, no two sentences were associated with the same base sentence. In total, 9 sentence lists were constructed. To prevent explicit attention to the critical word position, we constructed 54 incongruent filler sentences with a violation occurring equally in the 8^th^, 9^th^ or 10^th^ position of the sentence. The length of the filler sentences was also 11 words. Eighteen filler sentences were pseudo-randomly selected and added to each of the above lists. Within each list, different versions of the sentences were randomly intermixed to balance the presentation order.

### Procedure

The E-Prime software package (Psychology Software Tools, Pittsburgh, PA; version 1.1) was used for stimulus presentation and response collection. For each trial, subjects were asked to fixate on a centrally located asterisk that was presented for 500 ms. A sentence was then presented word by word. Each word was shown for 300 ms and followed by a 300 ms blank screen before the next word was displayed.

Three types of tasks were used. One was a reading task in which subjects were asked to silently read the sentence to establish activity related to comprehension. The SEM required subjects to make semantic congruency judgments, which is an explicit language task. After a sentence was presented, subjects were required to indicate whether it was semantically congruent or incongruent as fast and as accurately as possible by pressing a button within a 4-s response window. The FONT required subjects to make a judgment whether the font size changed between trials; this is an implicit language task. In the FONT task, a 500-ms cross fixation point was displayed following the sentence presentation and was replaced by a Chinese word ‘??’ (this Chinese word means “test”). Subjects were asked to judge whether this word appeared in the same size font as the words in the preceding sentence by pressing a button, which was also within a 4-s response window. In each of these three tasks, sentences were always presented in 40-point Times New Roman font, and the probe word was of the same font style but was either larger or smaller in size (50-point or 34-point). The font size was determined based on pilot studies for the appropriate level of task difficulty to avoid ceiling and flooring effects. We assessed both response accuracy and response time as appropriate. Responses given after the 4-s window had passed were considered errors. The response keys were counterbalanced across the subjects. An event-related design was used within each task. For the READ and SEM tasks, we selected three different inter-stimulus intervals (ISI): 2200 ms, 3200 ms, and 4200 ms; this yielded an averaged ISI of 3200 ms. For the FONT task, we added an additional 500 ms between the end of the sentences and the probe. The purpose of this was to maintain the same duration for each trial of the FONT task as those in the other two tasks. In the presentation phase of each trial, we used three ISIs: 1700 ms, 2700 ms, and 3700 ms for the READ, SEM, and FONT tasks, respectively. This yielded an average ISI of 2700 ms across all tasks. Each run was thus 19 min and 54 s in duration, including two 1-min rest periods.

Each subject completed three sessions of fMRI scans; one scan was conducted for each task. The reading task was always presented in the first session, but the order of the other two sessions was counterbalanced to prevent contamination of the reading task. For example, subjects may attempt to evaluate the semantic congruency of a sentence or attend to the font size, even if neither is required for the reading task. Subjects completed 10 practice trials using extra materials before each session. Except for in the reading task, subjects were provided with feedback related to their responses to ensure that they understood the task. No feedback was provided regarding any task during the fMRI scans. In the present study, we only analyzed the fMRI data that corresponded to both the SEM and FONT tasks; both of these tasks required manual responses. This was consistent with our aim to compare the network properties observed in explicit and implicit language tasks.

### BOLD-fMRI data acquisition

Subjects were asked to lie in a supine position inside the MRI scanner while wearing MRI-compatible earphones and goggles (Resonance Technology Company, Los Angeles, CA) and holding a button box. They were told not to move their head (which was restrained by padding) while inside the MRI scanner, but they could close their eyes for a short rest between two successive runs.

Image acquisition was performed using a 1.5 T Siemens Avanto MRI scanner. BOLD-fMRI data were acquired using a T2*-weighted gradient-echo echo-planar imaging (EPI) sequence with the following parameters: repetition time (TR)  =  2000 ms, echo time (TE)  =  43 ms, flip angle  =  90°, slice thickness  =  5 mm without gap, 26 interleaved transverse slices in ascending order covering the whole-brain, and voxel size  =  3.6×3.6×5 mm^3^. In addition, we also acquired 3D high resolution brain structural images with a T1-weighted MP-RAGE sequence (TR  =  11 ms; TE  =  3.3 ms; slice thickness  =  1 mm; voxel size  =  1×1×1 mm^3^, and 192 sagittal slices).

### Data preprocessing

The fMRI dataset of any subject was excluded if head motion was greater than 2 mm of displacement or 2° of rotation in any direction. Five patient datasets were removed because the top part of the cortex was not visualized in the MRI scan, and another dataset was removed due to a large head motion artifact (> 2 mm). Thus, twenty-one subjects were retained for further analysis.

**Figure 1:**
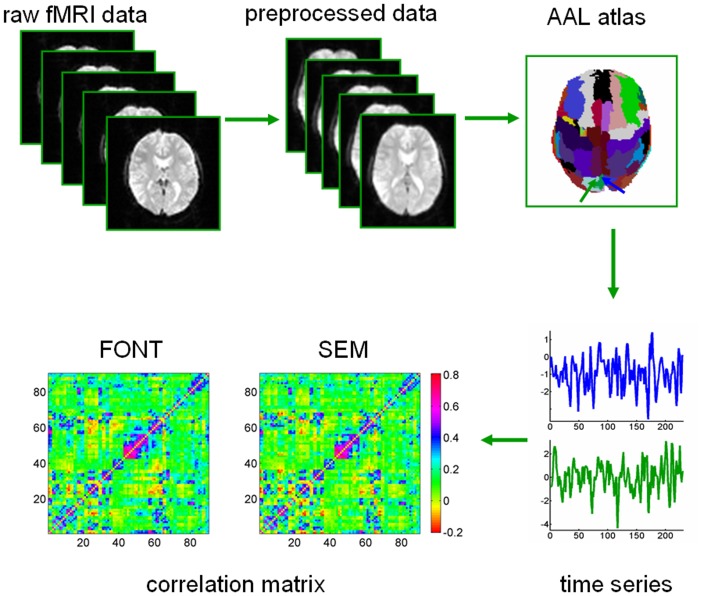
Illustration of the procedures used to construct brain functional networks. Raw functional MR images are preprocessed to produce normalized data that are further parcellated by a prior brain atlas into 90 brain regions. Then we averaged the time series over all voxels in each subject for each language task to generate the regional representative time course. The Pearson’s correlations between all possible pairs of 90 time courses for each specific task is computed and averaged for the same task for each subject. A connectivity matrix for a subject is shown for the explicit (SEM) and implicit (FONT) language tasks, respectively. The axial three-dimensional image of the template is shown using MRIcroN software (http://www.sph.sc.edu/comd/rorden/mricron/).

The fMRI datasets related to the FONT and SEM tasks were selected and preprocessed with SPM8 (http://www.fil.ion.ucl.ac.uk/spm/) and DPARSF [34]. For each subject, we discarded the first ten volumes to allow for MR signal equilibrium. The remaining images were first corrected for the acquisition time delay between slices within the same TR and were then realigned to the first volume to correct for inter-TR head motion. This realigning calculation provided a record of head motion within each fMRI scan. For each subject, the fMRI datasets were then spatially normalized based on the segmentation of the T1-weighted 3D brain structural images to the standard MNI-152 template; they were then resampled at a voxel size of 3×3×3 mm^3^. The waveform for each voxel was obtained through a 0.01 Hz high-pass filter to reduce the effects of low-frequency drift and high-frequency physiological noise. Figure 1 presents a schematic of the data analysis.

### Network analyses

Prior to performing the network analysis, we assessed the brain activation during each sentence condition against baseline levels of activation. We found similar brain activation patterns in widespread regions, including the left STG/MTG, left FFG, left precentral gyrus (PCG), left IFG, and bilateral occipital gyri at the *p* < 0.005 level (uncorrected). In a previous paper [12] that used the same dataset, we reported monotonic brain responses that increased from HC to LC and to SV in both tasks, especially in the left IFG. These results suggested that similar brain regions supported both explicit and implicit language tasks with varying amplitude. Because few trials were used (24 trials) in each condition and similar brain activation patterns were observed for each condition versus the baseline, we combined the HC, LC and SV conditions in each task to perform the network analysis.

The human brain functional networks were constructed according to the widely used automated anatomical labeling (AAL) brain atlas [35], which parcels the brain into 90 cortical and subcortical regions. Table 2 lists these brain regions and their abbreviations. We calculated the time series of each brain region by averaging the time courses of all voxels within a corresponding region. We also performed a linear regression to remove the effects of the following nuisance covariates: 1) the global mean signal, 2) the white matter signal, 3) the CSF signal, and 4) three translation and three rotation parameters of head motion. Then, we used the residuals of these time series for each brain region to calculate the Pearson’s correlation coefficient between each pair of brain regions. Thus, we obtained a 90×90 symmetrical connectivity matrix for each task in each subject. We applied a threshold to every element of the 90×90 connectivity matrix to obtain a binarized connectivity matrix for each subject. For these calculations, the sparsity, which is the ratio of the number of existing edges over the maximum possible number of edges, was used as the threshold to ensure that the networks corresponding to each subject had the same minimum number of edges. The binary connectivity matrix was represented by an undirected network by setting each brain region as a node and the using the inter-regional Pearson’s correlation as the edge. The topological properties of the networks were then analyzed according to graph theory.

**Table 2:** Brain regions used in constructing the human brain functional networks in the present study.

Index	Regions	Abb.	Index	Regions	Abb.
(1,2)	Precentral gyrus	PreCG	(47,48)	Lingual gyrus	LING
(3,4)	Superior frontal gyrus (dorsal)	SFGdor	(49,50)	Superior Occipital gyrus	SOG
(5,6)	Orbitofrontal cortex (superior)	ORBsup	(51,52)	Middle occipital gyrus	MOG
(7,8)	Middle frontal gyrus	MFG	(53,54)	Inferior occipital gyrus	IOG
(9,10)	Orbitofrontal cortex (middle)	ORBmid	(55,56)	Fusiform gyrus	FFG
(11,12)	Inferiorfrontal gyrus (opercular)	IFGoperc	(57,58)	Postcentral gyrus	PoCG
(13,14)	Inferiorfrontal gyrus (triangular)	IFGtriang	(59,60)	Superior parietal gyrus	SPG
(15,16)	Orbitofrontal cortex (inferior)	ORBinf	(61,62)	Inferior parietal lobule	IPL
(17,18)	Rolandic operculum	ROL	(63,64)	Supramarginal gyrus	SMG
(19,20)	Supplementary motor area	SMA	(65,66)	Angular gyrus	ANG
(21,22)	Olfactory	OLF	(67,68)	Precuneus	PCUN
(23,24)	Superior frontal gyrus (medial)	SFGmed	(69,70)	Paracentral lobule	PCL
(25,26)	Orbitofrontal cortex (medial)	ORBmed	(71,72)	Caudate	CAU
(27,28)	Rectus gyrus	REC	(73,74)	Putamen	PUT
(29,30)	Insula	INS	(75,76)	Pallidum	PAL
(31,32)	Anterior cingulate gyrus	ACG	(77,78)	Thalamus	THA
(33,34)	Middle cingulate gyrus	MCG	(79,80)	Heschl gyrus	HES
(35,36)	Posterior cingulate gyrus	PCG	(81,82)	Superior temporal gyrus	STG
(37,38)	Hippocampus	HIP	(83,84)	Temporal pole (superior)	TPOsup
(39,40)	Parahippocampal gyrus	PHG	(85,86)	Middle temporal gyrus	MTG
(41,42)	Amygdala	AMYG	(87,88)	Temporal pole (middle)	TPOmid
(43,44)	Calcarine cortex	CAL	(89,90)	Inferior temporal gyrus	ITG
(45,46)	Cuneus	CUN			

These regions are originally described in the Automated Anatomical Labeling (AAL) template by Tzourio-Mazoyer et al. (2002), and the abbreviations are listed according to Salvador et al. (2005) and Achard et al. (2006). The same 45 brain regions were extracted from the right and left hemispheres to provide 90 regions in total for each subject.

Note: Abb., abbreviations.

**Network parameters.** Graph theory was used to analyze the topological properties of the human brain functional networks [31], [36], [37]. We characterized the global topological organization of the brain networks by using the following parameters: the clustering coefficient, 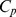, characteristic path length, 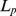, global efficiency, 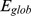, and local efficiency, 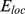. Their definitions and descriptions are listed in Table 3 [38]. 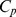 and 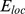 quantify the extent of local efficiency of information transfer within a network, while 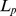 and 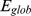 measure the global communication efficiency of a network.

**Table 3:** Definitions and descriptions of the global and regional parameters of brain functional networks used in the current study.

Network properties	Definitions	Descriptions
Global parameters	Cluster coefficient 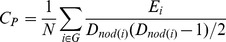	Given a network *G*(*N, M*) with *N* nodes and *M* edges, 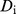 is the degree of node 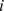 . 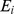 is the number of edges in 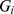 , the subgraph consisting of the neighbors of node 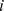 .
	Characteristic path length 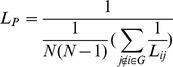	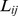 is the shortest path length between nodes 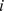 and 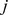 .
	Global efficiency 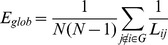	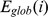 is the global efficiency of 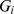 .
	Local efficiency 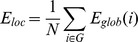	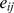 is the (*i, j*)th element in the formerly obtained binarized correlation matrix.
Nodal parameters	Betweenness centrality 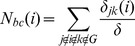	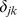 is the number of shortest paths from node 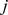 to node 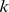 , and 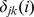 is the number of shortest paths from node 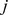 to node *k* that pass through the node 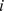 within graph 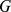 .

A small-world network can be characterized by the normalized clustering coefficient, 
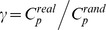
, and the normalized characteristic path length, 
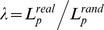
 [39]. Typically, a small-world network meets the following criteria: 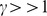 and 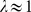 [39], or 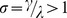. Here 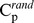 and 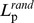 represent the means of the corresponding indices that were derived from the matched random networks (100 random networks were created in the calculation) created using a modified Maslov's wiring program, which preserved the same number of nodes, edges, and degree distribution as the real brain networks obtained from actual subjects.

We used the betweenness centrality (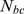) to characterize regional properties of the human brain functional networks. Its definition and description is also listed in Table 3 [38]. 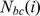 measures the influence of node 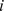 over the information flow between other nodes of the entire network and is the most widely used parameter to quantify the nodal centrality in neural networks.

**Integrated network parameters.** We used a series of sparsities as different threshold values to construct binary networks. Considering that selecting different threshold values could potentially cause changes in small-world network parameters, we used a threshold-independent network assessment by calculating the area under the sparsity curves of for each of the global and nodal network metrics [41]. The integrated network metrics were used for further analysis.

The integrated global parameters were defined as



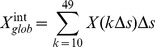
(1)


where 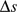 is the sparsity interval of 0.01, and 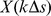 is one of the network parameters (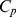,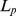,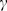,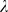,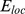, and 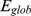) at the sparsity of 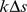. Similarly, the integrated regional nodal parameters may be calculated by



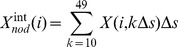
(2)


where 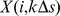
represents any nodal parameter (in this example, it is the betweenness centrality 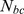) of node 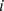 at the sparsity *kΔs*. The topological properties of the obtained networks could also be affected by the choice of sparsity values. After the calculation, we found that in a range of sparsities (0.10 ≤ sparsity ≤ 0.49) at the interval of 0.01, the brain functional networks of all of the subjects exhibited prominent small-world characteristics.

**Hub identification.** Hubs play important roles in the brain functional networks. Hubs refer to the nodes that are connected to the greatest number of other nodes in the network [42]. In the small-world network, a small proportion of regions (hub) have a large proportion of connection, while a larger proportion of regions (non-hub) have a small proportion of connection. To determine the hubs of the brain functional networks, we followed previous studies [43], [44] and calculated the normalized betweenness centrality for each node as follows:



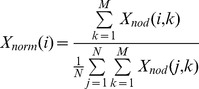
(3)


where 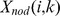 represents the integrated nodal betweenness centrality at node *i* for the network of subject *k*, *N* the number of nodes, and *M* the number of subjects.

For a network, an important characteristic is the efficiency of information transfer among the network nodes. In general, network hubs exhibit high betweenness centrality. To this end, the present analyses identified the node *i* as a hub of the network if the normalized betweenness centrality satisfies the criterion: 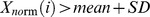, where 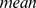 stands for the mean value and 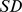 for the standard deviation of 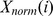 across all subjects per task [44].

### Statistical analyses

A *t*-test was used to detect the differences in the integrated network parameters of the brain functional networks between the SEM and FONT tasks. The threshold was *p* < 0.05 (FDR correction). Notably, before the *t-*test was conducted, we constructed Lilliefors test [45] to explore whether the parameters in our study followed a normal distribution. We found that the related parameters were normally distributed.

## Results

### Behavioral performance

In the explicit language task, we found no significant main effect of the sentence type in terms of accuracy (HC, LC and SV: 93.6±9.1%, 91.2±7.3%, 93.9±4.2%, respectively; *F* < 1), but we did detect a significant main effect of the sentence type in terms of reaction time (RT) (mean ± SD  =  764±341 ms, 803±426 ms, 584±307 ms, *F* (2, 40)  =  10.2, *p* < 0.001, HC  =  LC > SV). These results are similar to those of our previous studies [12], [17]. In the implicit language task, the significant condition difference was neither found in terms of accuracy (HC: 83.3±19.4%; LC: 83.9±21.3%; SV: 86.1±17.9%; *F* < 1) nor in RT (HC: 1037±293 ms; LC: 1034±385 ms; SV: 1011±268 ms; *F* < 1). The results of the present study indicated that the behavioral performances have not been explicitly interfered with by the three different conditions that are manipulated in the implicit language task.

### Small-world properties

**Figure 2:**
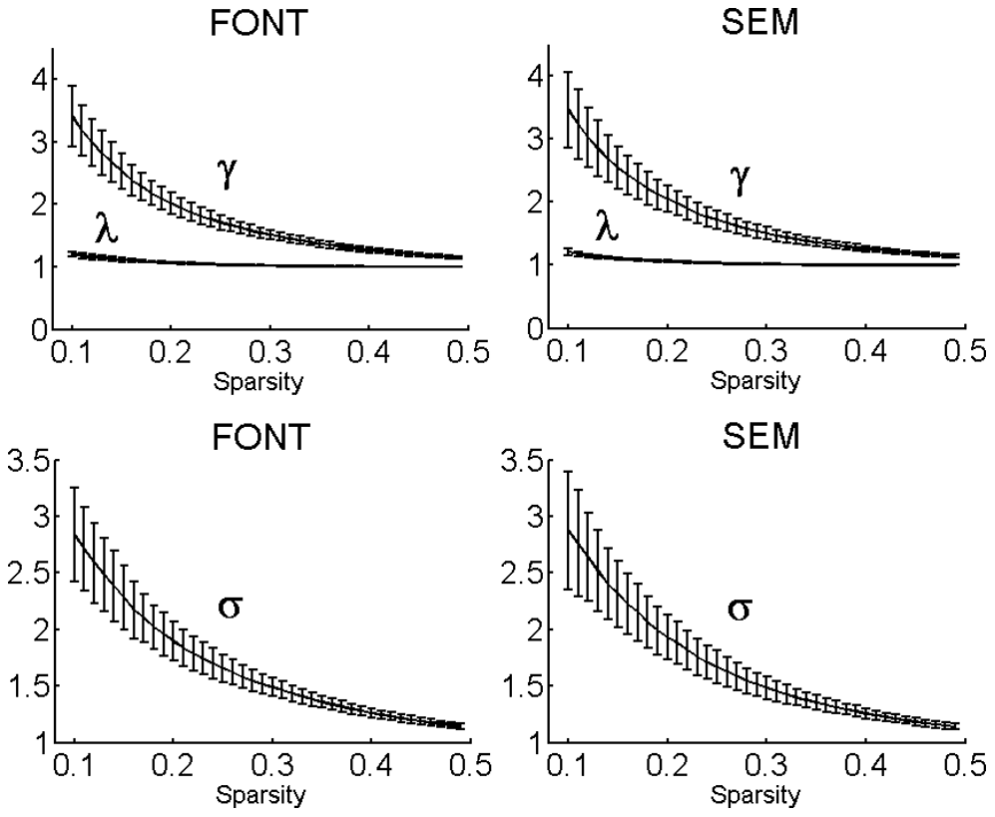
Small-world properties changing with the varied sparsity of the functional networks for both the explicit and implicit language tasks. Here 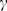 stands for the normalized clustering coefficient, 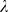 for the normalized characteristic path length, and σ for the ratio of 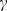 to 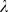. The values of 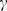 and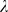 were evaluated on each individual brain network and then averaged over all subjects in the explicit and implicit language tasks, respectively. In a wide range of sparsity (0.10 ≤ sparsity ≤ 0.49), the functional networks for the implicit or explicit language tasks exhibit 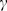
>1, 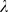
≈1, and σ> 1.1, which indicated prominent small-world properties.

We analyzed the topological properties of the brain functional networks in the sparsity range from 0.10 to 0.49. We obtained γ>>1, 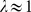, and σ >1.1 over the range of 0.10 ≤ sparsity ≤ 0.49 for each of the implicit and explicit language tasks (Figure 2); these terms demonstrated that the brain functional networks in both tasks exhibited prominent small-world properties.

Table 4 lists the statistical comparison of the integrated global network parameters between the explicit and implicit language tasks. We found significantly greater local efficiency and an increased clustering coefficient in the implicit language task (*p* < 0.05), as well as a trend of increasing global efficiency and shortest path length in the explicit language task. No effect of task was detected on the integrated clustering coefficient or the integrated weighted shortest path length.

**Table 4:** Integrated global parameters mean (SD) of the human brain functional networks and their statistical difference between the explicit and implicit language tasks.

Parameters	Implicit language task	Explicit language task	*t*-value	*p*-value
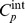	0.225 (0.010)	0.220 (0.011)	2.46	0.02
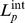	0.654 (0.013)	0.652 (0.013)	1.27	0.22
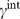	0.676 (0.045)	0.679 (0.055)	0.26	0.80
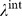	0.406 (0.003)	0.405 (0.003)	1.52	0.14
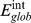	0.239 (0.003)	0.240 (0.003)	1.72	0.10
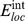	0.299 (0.005)	0.297 (0.005)	3.01	0.007

Note: 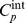,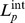,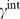,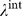,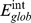, and 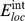 correspond to the integrated clustering coefficient, integrated characteristic path length, integrated normalized clustering coefficient, integrated normalized shortest path length, integrated global efficiency, and integrated local efficiency, respectively.

### Network hubs

In this study, we found that the hub regions were mainly located in the association cortices (Table 5); there were 12 hubs related to the implicit language task and 17 hubs related to the explicit language task. Among them, ten were common hubs shared in the functional networks corresponding to both tasks, which included the bilateral supplementary motor area (SMA), bilateral median cingulate (MCG), bilateral middle temporal gyrus (MTG), left middle occipital gyrus (MOG.L), left fusiform gyrus (FFG.L), right superior temporal gyrus (STG.R), and right superior temporal gyrus (TPOsup.R). Two brain regions were identified as hubs specific to the implicit language task: the left superior temporal gyrus (STG.L) and right inferior temporal gyrus (ITG.R). Meanwhile, we identified 7 brain regions, which were hubs specific to the explicit language tasks: the left precentral gyrus (PreCG.L), PreCG.R, right medial orbitofrontal cortex (ORBmed.R), right parahippocampal gyrus (PHG.R), FFG.R, right precuneus (PCUN.R), and left superior temporal gyrus (TPOsup.L).

**Table 5:** Hub regions of the brain functional networks corresponding to the explicit and implicit language tasks, respectively.

Region	Classification	Normalized betweenness centrality
		Implicit language task	Explicit language task
PreCG.L	Primary	-	1.77
PreCG.R	Primary	-	1.79
SMA.L	Association	1.96	1.54
SMA.R	Association	1.73	1.6
ORBmed.R	Paralimbic	-	1.65
MCG.L	Paralimbic	2.02	1.78
MCG.R	Paralimbic	1.85	1.75
PHG.R	Paralimbic	-	1.65
MOG.L	Association	1.88	1.53
FFG.L	Association	2.27	2.06
FFG.R	Association	-	1.53
PCUN.R	Association	-	1.6
STG.L	Association	2.46	-
STG.R	Association	2.63	2.19
TPOsup.L	Paralimbic	-	1.83
TPOsup.R	Paralimbic	2.57	1.9
MTG.L	Association	2.22	2.19
MTG.R	Association	2.26	2.79
ITG.R	Association	2.19	-

Note: “–” indicates that the value of the normalized betweenness centrality in the region was within one standard deviation from the mean. The shaded texts were the shared hub regions detected under both the two tasks.

### Task related changes in betweenness

**Figure 3:**
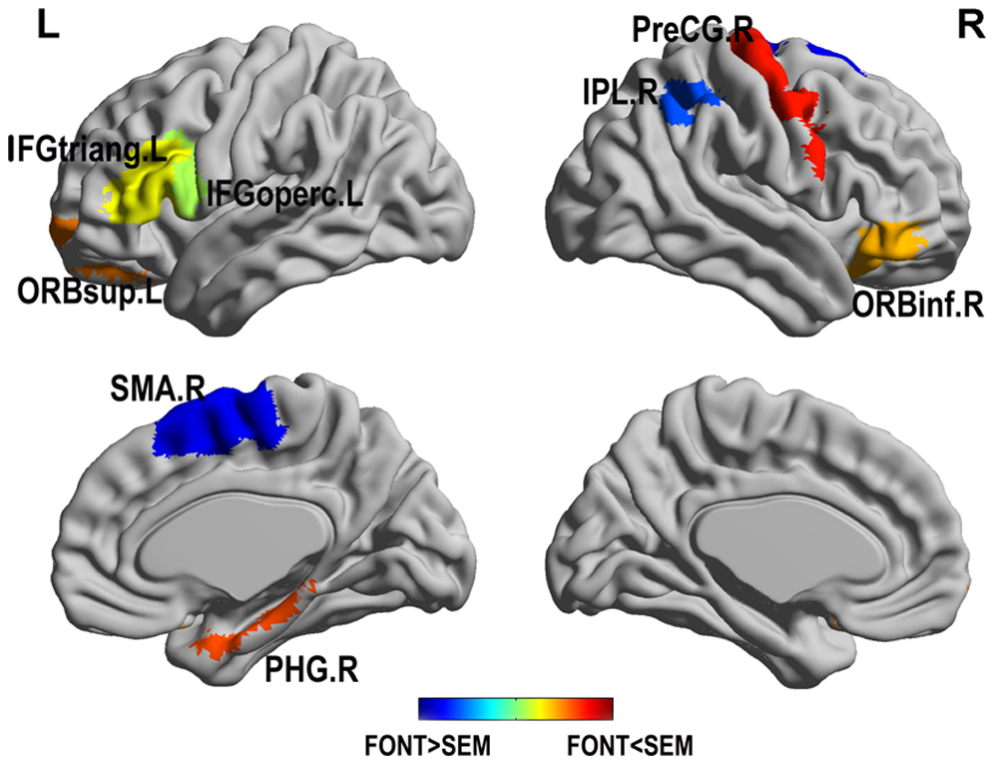
Brain regions exhibited significant alterations in the integrated betweenness centrality of the functional networks between the explicit and implicit language tasks. Regions color-coded in cold (warm) represent the increased (decreased) value of integrated betweenness centrality in the implicit language task compared to the explicit language task. Abbreviations: L, left hemisphere; R, right hemisphere.

Table 6 lists the brain regions that exhibited significant between-task differences in the integrated betweenness centrality of the functional networks. Compared with the implicit language task, the brain networks corresponding to the explicit language task demonstrated significantly reduced integrated betweenness centrality in the SMA.R and IPL.R and significantly increased integrated betweenness centrality in the PreCG.R, ORBsup.L, left opercular inferior frontal gyrus (IFGoperc.L), left triangular inferior frontal gyrus (IFGtriang.L), ORBinf.R, and PHG.R. These brain regions were rendered on the cortical surface and are shown in Figure 3.

**Table 6:** Brain regions showing significant difference in the mean (SD) integrated betweenness centrality between the brain functional networks corresponding to the explicit and implicit language tasks.

Region	Classification	Implicit language task	Explicit language task	t-value (p-value)
**Implicit > Explicit**				
SMA.R	Association	0.717 (0.454)	0.574 (0.303)	2.74(0.013)
IPL.R	Association	0.370 (0.149)	0.288 (0.150)	2.58(0.018)
**Implicit < Explicit**				
PreCG.R	Primary	0.507 (0.226)	0.650 (0.335)	2.28(0.034)
ORBsup.L	Paralimbic	0.221 (0.111)	0.345 (0.183)	2.81(0.011)
IFGoperc.L	Association	0.306 (0.159)	0.416 (0.223)	2.93(0.008)
IFGtriang.L	Association	0.220 (0.107)	0.305 (0.145)	2.58(0.018)
ORBinf.R	Paralimbic	0.373 (0.214)	0.508 (0.315)	2.34(0.030)
PHG.R	Paralimbic	0.315 (0.224)	0.433 (0.310)	2.53(0.020)

## Discussion

In the present study, we examined the topological properties of brain functional networks involved in explicit and implicit language tasks. These networks demonstrated prominent small-worldness. We also observed widespread common hubs in occipital, temporal, and frontal regions corresponding to both the implicit and explicit language tasks. Moreover, we found between-task differences of the global network parameters and of betweenness centrality in key language regions. The details of these findings and their implications are discussed below.

For the first aim of the study, we investigated whether the whole brain functional networks shared common properties in both implicit and explicit language tasks, regardless of task-specific effects. We found that the brain functional networks corresponding to both explicit and implicit language tasks exhibited small-worldness, regardless of task-specific effects. Small-worldness indicates that the networks with high clustering coefficients and short path lengths [39]. In our study, it reflects the need of the brain networks to satisfy the competitive demands of local and global processing in both language tasks. Optimal brain function requires balance between local specialization and global integration of brain functional activity [46]. Furthermore, we noticed that several regions such as bilateral MTG and right STG shared across both implicit and explicit tasks have been previously reported in studies of sentence comprehension. The sentence comprehension network that we found is also consistent with that reported by previous studies [20], [21] of explicit language task.

Furthermore, we found that common hubs in widespread regions supported the semantic processing in both the implicit and explicit tasks. Hubs of the networks for both tasks were identified in the bilateral SMA, bilateral MCG, bilateral MTG, MOG.L, FFG.L, STG.R, and TPOsup.R, which were consistently engaged in language related processes [1]–[4], [47], [48]. Previous studies have indicated that the temporal lobe is engaged in semantic representation storage and access [2], [5], the FFG is a key region involving in word recognition and semantic processing [2], [49], [50], and the MTG is a region sensitive to both semantic access [5] and semantic control [5], [51], [52]. In addition, several previous studies found that the cingulate cortex may contribute to semantic control during sentence comprehension [17], [53], [54] or to general cognitive control processes [55], and the occipital region was involved in the sentence comprehension when hard or odd sentences were presented to support visual function [17], [56]. These results were consistent with previous findings in explicit language task contexts [19]–[21], suggesting that large common network supports sentence comprehension in both explicit and implicit language tasks.

For the second aim of the study, we asked whether and how tasks modulate network properties. We found that the brain functional networks involved in explicit and implicit language exhibited significantly reduced levels of local efficiency while an increasing trend in global efficiency in the explicit language task compared with the implicit language task (Table 4). The differences in local and global efficiency between the two language tasks may due to the fact that the optimized brain requires a suitable balance between local specialization and global integration of brain activity [46]. As the global efficiency is a measure of the information transfer in the brain, whereas local efficiency is a measure of the information exchange of each sub-area of the brain [57], the differences in both the global and local efficiency between these two tasks may reflect the brain optimization in different language tasks. In the explicit language task, subjects must participate in semantic retrieval, semantic integration, and semantic control to elaborately analyze semantic information. While in the implicit language task, subjects were required to visually match the font size as part of a feature detection paradigm. This means that performance of the explicit language task relies on information transfer across widespread regions of the brain (such as IFG, MTG, STG etc). As a consequence, the explicit language task should involve higher levels of global efficiency than the implicit language task, which in turn made the local efficiency lower than the implicit language task.

Furthermore, we also found between-task differences in the betweenness centrality in varying language tasks (Table 6). The integrated betweenness centrality was higher in the left IFG (IFGoperc.L and IFGtriang.L) during the explicit language than that in the implicit language task. The left IFG is an area of the brain that is important for language comprehension [2], [3], [48], [58]. Particularly, several previous studies have indicated that the IFGoperc.L and IFGtriang.L are important for elaborated semantic analysis [3], [12], [15]–[17], [59]–[62], such as semantic candidate selection and inhibition.

However, the different results between two task conditions obtained in the present study may reflect a mixture of both semantic and non-semantic task-specific processes. For example, we observed extra hubs in the explicit language task compared with the implicit language task. Previous studies have found that activation of the PCG is associated with phonological rehearsal [63] and that activation of the ORBmed.R and PCUN.R corresponds to the theory of mind analyses [54], [64]. These results demonstrate that neural semantic processing in explicit language tasks is more widespread than that observed in implicit language tasks [65]. Moreover, we also found increased integrated betweenness centrality in the SMA.R and IPL.R during the implicit language task. As the subjects were asked to make font size judgments, they may need to maintain representations of the font size of the sentence words in working memory to compare with those of the probe. Thus, the IPL.R which contribute to working memory [66] and attention control [67] was engaged in the implicit language task. Additionally, the SMA and pre-SMA were associated with motor function such as response preparing [68]; the observed difference in betweenness centrality of the SMA.R may due to poorer performance in the implicit language task, as the accuracy was much lower and the RT was longer in this task than those in the explicit language task.

It should be noted that the left IFG was not recognized as a hub for language processing in the present study. Although the activation in the left IFG was often reported in sentence comprehension studies [2], [3], [69], there was a report showing lack of activation in left IFG when bilateral temporal regions activation was involved [6]. It’s possible that the left IFG contributes to effortful semantic control but is not necessary participating in the simple sentence comprehension. Yet, with the dataset, we did found enhanced BOLD signal change in the left IFG in simple sentence comprehension relative to fixation baseline in the two language tasks [12]. These results made the explanation less likely relevant to the left IFG.

Interestingly, many prior studies did not identify the IFG as a network hub [20], [43], [44], [57], [70]. For example, Ye et al. [20] used a graph theoretical approach to reveal network activity during sentence comprehension, and did not identify the IFG as a hub. One may speculate whether these results were due to the selected AAL template was based on single person’s brain. We also used the Harvard-Oxford atlas (HOA) to test the probability that the left IFG would be recognized as hub. The HOA was generated from a probabilistic atlas of the Harvard-Oxford Structural Atlas (http://neuro.debian.net/pkgs/fsl-harvard-oxford-atlases.html) that defines regions (see Table S1, supplemental material) based on standard anatomical boundaries (probability threshold  =  25%). The results of this analysis revealed similar between-task differences in network parameters, such as global and local efficiency, as did the AAL (see Table S2, supplemental material). We also found similar hubs (see Table S3, supplemental material) and regions which indicated task differences in terms of betweenness centrality (see Table S4, supplemental material). Nevertheless, the left IFG was not recognized as hub. Additional reasons may be the differences in nodes and edges definition which may influence the calculated network properties [41], [71]–[75].

In conclusion, the present study revealed that the human brain functional networks involved in sentence comprehension met small-worldness criteria in both explicit and implicit language tasks. The task related differences in the network properties indicated different effects of the explicit and implicit language tasks on the brain functional networks. These findings increase understanding of the neural basis of language comprehension.

## Supporting Information

File S1 Table S1. Brain regions used in constructing the human brain functional networks in the present study. The HOA atlas is generated from a probabilistic atlas of Harvard-Oxford Structural Atlas that defines regions based on standard anatomical boundaries (probability threshold  =  25%). Table S2. Integrated global parameters of the human brain functional networks and the statistical difference in the integrated global parameters between the implicit and explicit language tasks (Mean ± SD). Table S3. Hub regions of the brain functional networks corresponding to the implicit and explicit language tasks. Table S4. Brain regions showing significant difference in the integrated betweenness centrality between the brain functional networks corresponding to the implicit and explicit language tasks.
(DOC)
