# Supplementary material for: Large Scale Brain Functional Networks Support Sentence Comprehension: Evidence from Both Explicit and Implicit Language Tasks
Source: PLoS One. 2013 Nov 11;8(11):e80214. doi: 10.1371/journal.pone.0080214 (PMC3823842; doi:10.1371/journal.pone.0080214)
Supplement: File S1 — Table S1. Brain regions used in constructing the human brain functional networks in the present study. The HOA atlas is generated from a probabilistic atlas of Harvard-Oxford Structural Atlas that defines regions based on standard anatomical boundaries (probability threshold = 25%). Table S2. Integrated global parameters of the human brain functional networks and the statistical difference in the integrated global parameters between the implicit and explicit language tasks (Mean ± SD). Table S3. Hub regions of the brain functional networks corresponding to the implicit and explicit language tasks. Table S4. Brain regions showing significant difference in the integrated betweenness centrality between the brain functional networks corresponding to the implicit and explicit language tasks. (DOC) [file pone.0080214.s001.doc]

**Table S1**

| Index | Regions | Abb. | Index | Regions | Abb. |
| --- | --- | --- | --- | --- | --- |
| (1,2) | Frontal Pole | FP | (57,58) | Cingulate Gyrus, anterior division | CGa  ACG |
| (3,4) | Insular Cortex | INS | (59,60) | Cingulate Gyrus, posterior division | CGp  PCG |
| (5,6) | Superior Frontal Gyrus | SFG | (61,62) | Precuneus Cortex | PCN |
| (7,8) | Middle Frontal Gyrus | MFG | (63,64) | Cuneal Cortex | CN |
| (9,10) | Inferior Frontal Gyrus, pars triangularis | IFGtriang | (65,66) | Frontal Orbital Cortex | FOC |
| (11,12) | Inferior Frontal Gyrus, pars opercularis | IFGoperc | (67,68) | Parahippocampal Gyrus, anterior division | PHGant |
| (13,14) | Precentral Gyrus | PreCG | (69,70) | Parahippocampal Gyrus, posterior division | PHGpost |
| (15,16) | Temporal Pole | TPO | (71,72) | Lingual Gyrus | LING |
| (17,18) | Superior Temporal Gyrus, anterior division | STGant | (73,74) | Temporal Fusiform Cortex, anterior division | FFGant |
| (19,20) | Superior Temporal Gyrus, posterior division | STGpost | (75,76) | Temporal Fusiform Cortex, posterior division | FFGpost |
| (21,22) | Middle Temporal Gyrus, anterior division | MTGant | (77,78) | Temporal Occipital Fusiform Cortex | FFGtempoccip |
| (23,24) | Middle Temporal Gyrus, posterior division | MTGpost | (79,80) | Occipital Fusiform Gyrus | FFGoccip |
| (25,26) | Middle Temporal Gyrus, temporooccipital part | MTGtempoccip | (81,82) | Frontal Operculum Cortex | FO |
| (27,28) | Inferior Temporal Gyrus, anterior division | ITGant | (83,84) | Central Opercular Cortex | CO |
| (29,30) | Inferior Temporal Gyrus, posterior division | ITGpost | (85,86) | Parietal Operculum Cortex | PO |
| (31,32) | Inferior Temporal Gyrus, temporooccipital part | ITGtempoccip | (87,88) | Planum Polare | PP |
| (33,34) | Postcentral Gyrus | PoCG | (89,90) | Heschl's Gyrus | H |
| (35,36) | Superior Parietal Lobule | SPL | (91,92) | Planum Temporale | PT |
| (37,38) | Supramarginal Gyrus, anterior division | SMGant | (93,94) | Supracalcarine Cortex | SCLC |
| (39,40) | Supramarginal Gyrus, posterior division | SMGpost | (95,96) | Occipital Pole | OP |
| (41,42) | Angular Gyrus | AG | (97,98) | Brain-Stem | Bst |
| (43,44) | Lateral Occipital Cortex, superior division | LOCsup | (99,100) | Thalamus | Thal |
| (45,46) | Lateral Occipital Cortex, inferior division | LOCinfe | (101,102) | Left Caudate | Caud |
| (47,48) | Intracalcarine Cortex | CALC | (103,104) | Left Putamen | Put |
| (49,50) | Frontal Medial Cortex | FMC | (105,106) | Left Pallidum | Pall |
| (51,52) | Supplementary Motor Cortex | SMC  SMA | (107,108) | Left Hippocampus | HIP |
| (53,54) | Subcallosal Cortex | SC | (109,110) | Amygdala | AMYG |
| (55,56) | Paracingulate Gyrus | PAC | (111,112) | Accumbens | Accbns |

**Table S2**

| Parameters | HOA atlas | | | | AAL atlas | | | |
| --- | --- | --- | --- | --- | --- | --- | --- | --- |
|  | Implicit task | Explicit task | *t*-value | *p*-value | Implicit task | Explicit task | *t*-value | *p*-value |
|  | 0.191± 0.010 | 0.186 ± 0.007 | 2.89 | 0.009* | 0.225 ± 0.010 | 0.220 ± 0.011 | 2.46 | 0.02* |
|  | 0.624± 0.016 | 0.619 ± 0.015 | 1.70 | 0.105 | 0.654 ± 0.013 | 0.652 ± 0.013 | 1.27 | 0.22 |
|  | 0.598 ± 0.042 | 0.606 ± 0.053 | -0.70 | 0.494 | 0.676 ± 0.045 | 0.679 ± 0.055 | 0.26 | 0.80 |
|  | 0.367 ± 0.005 | 0.365 ± 0.003 | 1.87 | 0.076 | 0.406 ± 0.003 | 0.405 ± 0.003 | 1.52 | 0.14 |
|  | 0.204 ± 0.004 | 0.205 ± 0.004 | -1.95 | 0.065 | 0.239 ± 0.003 | 0.240 ± 0.003 | 1.72 | 0.10 |
|  | 0.258± 0.006 | 0.256 ± 0.005 | 2.75 | 0.012* | 0.299 ± 0.005 | 0.297 ± 0.005 | 3.01 | 0.007* |

**Table S3**

| HOA atlas | | |  | AAL atlas | | |
| --- | --- | --- | --- | --- | --- | --- |
| Region | Implicit task | Explicit task |  | Region | Implicit task | Explicit task |
| FP.L | 1.75 | 1.68 |  | PreCG.L | - | 1.77 |
| FP.R | 1.55 | - |  | PreCG.R | - | 1.79 |
| INS.L | 1.73 | 2 |  | SMA.L | 1.96 | 1.54 |
| INS.R | 1.84 | 1.69 |  | SMA.R | 1.73 | 1.6 |
| SFG.L | - | 1.54 |  | ORBmed.R | - | 1.65 |
| SFG.R | 1.85 | 1.72 |  | MCG.L | 2.02 | 1.78 |
| PreCG.L | 1.65 | 1.78 |  | MCG.R | 1.85 | 1.75 |
| PreCG.R | 1.62 | 1.6 |  | PHG.R | - | 1.65 |
| TPO.L | 2.57 | 2.16 |  | MOG.L | 1.88 | 1.53 |
| TPO.R | 3.27 | 3.02 |  | FFG.L | 2.27 | 2.06 |
| MTGtempoccip.L | 1.61 | 1.7 |  | FFG.R | - | 1.53 |
| MTGtempoccip.R | 1.66 | 1.81 |  | PCUN.R | - | 1.6 |
| LOCsup.L | 1.86 | 1.93 |  | STG.L | 2.46 | - |
| LOCsup.R | - | 1.51 |  | STG.R | 2.63 | 2.19 |
| ACG.L | 1.58 | - |  | TPOsup.L | - | 1.83 |
| PCN.R | 1.62 | - |  | TPOsup.R | 2.57 | 1.9 |
| FOC.L | 2.18 | 2.07 |  | MTG.L | 2.22 | 2.19 |
| FOC.R | - | 1.79 |  | MTG.R | 2.26 | 2.79 |
| Put.L | - | 1.57 |  | ITG.R | 2.19 | - |

**Table** S4

| HOA atlas | |  | AAL atlas | |
| --- | --- | --- | --- | --- |
| Region | *t*-value (*p*-value) |  | Region | *t*-value (*p*-value) |
| **Implicit > Explicit** |  |  | **Implicit > Explicit** |  |
| FFGoccip.R | 2.11 (0.048) |  | SMA.R | 2.74（0.013） |
|  |  |  | IPL.R | 2.58（0.018） |
|  |  |  |  |  |
| **Implicit < Explicit** |  |  | **Implicit < Explicit** |  |
| IFGtriang.L | 2.70 (0.014) |  | PreCG.R | 2.28（0.034） |
| IFGtriang.R | 2.16 (0.043) |  | ORBsup.L | 2.81（0.011） |
| IFGoperc.L | 2.21 (0.039) |  | IFGoperc.L | 2.93（0.008） |
| STGpost.R | 2.31 (0.032) |  | IFGtriang.L | 2.58（0.018） |
| ITGant.L | 2.27 (0.034) |  | ORBinf.R | 2.34（0.030） |
| CN.L | 2.35 (0.029) |  | PHG.R | 2.53（0.020） |
| FO.L | 2.59 (0.018) |  |  |  |
